# Trends in Healthy Life Years Between 2005 and 2019 in 31 European Countries: The Compression or Expansion of Morbidity?

**DOI:** 10.3389/ijph.2024.1607574

**Published:** 2024-10-16

**Authors:** Jakub Straka, Luděk Šídlo, Ivana Kulhánová

**Affiliations:** ^1^ Department of Demography and Geodemography, Faculty of Science, Charles University, Prague, Czechia; ^2^ Department of Social Geography and Regional Development, Faculty of Science, Charles University, Prague, Czechia

**Keywords:** healthy life years, population ageing, compression, expansion, dynamic equilibrium

## Abstract

**Objectives:**

Our objective was to assess morbidity trends in Europe and to classify European countries based on population ageing theories: the compression, expansion and dynamic equilibrium of morbidity.

**Methods:**

The proportions of healthy life years were calculated for 31 European countries for the period 2005–2019 based on life expectancy values and healthy life years at age 65 years adopted from the Eurostat database. European countries were classified according to morbidity patterns applying the standard deviation distance from the average of relative change method between the selected years.

**Results:**

A large degree of variation in terms of life expectancy and healthy life years at age 65 years was determined between 2005 and 2019. While the life expectancy differences between men and women were consistent across all the European countries, the gender gap concerning healthy life years was more diverse. Approximately one-third of the countries fell into the expansion, compression and dynamic equilibrium categories, respectively.

**Conclusion:**

Significant variations were identified in healthy life year trends across European countries, which underscores the need for preventive strategies.

## Introduction

European health systems vary significantly according to the historical, cultural, economic and political context of the respective country [1]. Although all the respective health systems have their strengths and challenges, the differing structures influence both demographic dynamics and health outcomes. All European health systems have developed mechanisms for addressing healthcare challenges, including population ageing [2].

Population ageing refers to the demographic shift towards an older population. In recent decades, European countries have registered increasing proportions of the elderly in society as a result of increasing life expectancy. Life expectancy is a summary measure of the health of the population that considers the prevailing social and economic conditions of the respective country [3, 4]. It has been shown that when life expectancy at birth exceeds 70 years, long-term chronic diseases exert a significant impact of the health and quality of life of the population [5].

Consequently, discussions have been held as to whether prolonging the human lifespan is not just adding years of illness. Three theories of the relationship between mortality and morbidity were formulated in the 1980s. Firstly, the theory of the compression of morbidity proposes that a reduction in mortality is accompanied by an improvement in the health status [6]. Fries assumed that the postponement of morbidity to higher ages leads to shorter time periods spent in poor health due to successful primary prevention and improved living conditions [7, 8]. The original idea of morbidity compression stated that the age of the onset of chronic illness can be postponed more than can the age at death, thus squeezing most of the morbidity in life into a shorter period with less disability over the life span, whereas a reviewed version emphasised the importance of healthy ageing in terms of improved health throughout the life cycle [9]. Secondly, the theory of the expansion of morbidity proposes that extra years are in most cases spent in poor health. This theory assumes that medical progress prolongs life with the presence of chronic diseases, thus leading to the expansion of morbidity [10–12]. Thirdly, the theory of dynamic equilibrium assumes that the proportion of morbidity neither increases or decreases in the added years of life, rather it is in balance, i.e., the increased prevalence of morbidity affects only less serious conditions [13]. This theory combines both perspectives by assuming that even with an increase in life expectancy, the relative number of life years spent in good health remains unchanged. Further, the quality of life is improved as a result of medical progress [13]. These three scenarios place differing demands on health services and systems. Thus, in terms of planning, it is of particular importance for national health services to assess the prevalent morbidity patterns.

Most studies reported in the international literature restrict the investigation of the various theoretical concepts of morbidity to a single country [5, 14–16] or the application of certain measures [16–18]. Kreft and Doblhammer (2016) investigated the expansion and compression of long-term care in Germany based on measuring the number of care-need-free life years. Health-adjusted life expectancy (HALE) has been employed to evaluate the compression and expansion of morbidity in Canada [16] and Europe [18]. Self-perceived health ratings have been applied to measure health changes in Austria [5] and disability-free life expectancy in the UK and EU-28 [19]. Only two recently published studies have investigated broader sets of European countries [18, 19]. However, both studies investigated changes over extended periods without analysing morbidity patterns in detail.

The social and economic burden of population ageing will, to a great extent, be defined by future trends in morbidity [5]. The results could, therefore, be important for policymakers in terms of improving the health of the population and securing sustainable healthcare services and pension funding [20]. The aim of this study is, thus, to examine whether additional life years are spent in good or in poor health based on the study of a broad set of European countries. Applying the healthy life years (HLY) indicator, which provides an important tool for the monitoring of the health trends of populations, we assessed the evidence for the validity of morbidity scenarios in a range of European countries. Further, the study provides a country typology according to the population ageing scheme, i.e., compression, expansion or the dynamic equilibrium of morbidity.

## Methods

We employed data on life expectancy and HLYs at age 65 years from the Eurostat database [21]. Life expectancy refers to the average number of years a person is expected to live and is calculated based on mortality tables that show the order of population extinction in individual countries. The mortality tables were constructed using information on the number of deaths and births and the population structure by age and sex provided by national statistical offices [22].

The HLY indicator is used widely for the measurement of the health of populations. This indicator adds a quality dimension to life expectancy by reflecting the number of remaining years available to a person of a certain age without significant usual activity limitations, as assessed by the Global Activity Limitation Instrument (GALI) [23]. GALI measures the presence of long-standing activity limitations. The calculation of HLYs is based directly on life expectancy and the GALI indicator applying the Sullivan method [24, 25], which was selected due to its lower input data requirements. The GALI data was obtained from the European Union Statistics on Income and Living Conditions (EU-SILC) survey [26], which poses the following questions to respondents “*For at least the past 6 months, to what extent have you been limited because of a health problem in activities people usually do? Would you say you have been: 1) severely limited; 2) limited but not severely; or 3) not limited at all?”* According to the Eurostat methodology, we considered those who selected the third option, “not limited at all” for the purpose of defining persons without significant limitations [27]. We obtained a complete time series of life expectancy and HLY at age 65 years by gender for the period 2005–2019 for 31 European countries for this study. In cases in which no HLY values were available from the Eurostat database for specific years or countries, we used the value from the preceding year in order to maintain the consistency of the analysis.

We calculated the proportion of HLY of the total life expectancy at age 65 years by gender for each year of the period 2005–2019. This proportion represents the share of remaining life expectancy that an individual at age 65 years can expect to live in good health, i.e., free of any significant activity limitations as assessed using GALI data. We subsequently analysed the relative change in this proportion over time across the various countries and by gender. The examination of these changes allowed us to determine whether or not the living conditions of the population improved or deteriorated over the studied period. More specifically, the analysis allowed us to categorise the trends in terms of the three considered scenarios: morbidity compression (an increase in the proportion of life spent in good health), morbidity expansion (a decrease in this proportion) and dynamic equilibrium (the proportion remained relatively stable). These scenarios provided insight into the broader public health dynamics in the European countries considered in the study.

The categorisation of the countries according to the morbidity compression, expansion or dynamic equilibrium scenarios involved the calculation of the mean and the standard deviation of the relative change in the proportion of healthy life years between the start and end years of the studied period (2005 and 2019) by gender. The limits of the intervals for each scenario were then determined based on the standard deviation from the mean, as shown in Table 1. This approach allowed for a statistically grounded categorisation approach via which the intervals reflected the natural variation in the data, which followed a normal distribution (Gaussian distribution) trend. By setting the interval boundaries applying multiples of the standard deviation (k_1_ = 1.0, k_2_ = 0.5), we aimed to capture significant deviations from the mean, thereby identifying those countries that experienced notable shifts in their morbidity trends. This method was chosen since it provides a clear and objective approach to classifying the countries based on their relative change in HLY. Although other methods were considered, this approach was selected due to its simplicity and effectiveness in terms of distinguishing between the considered morbidity scenarios, considering the aim of categorising the countries in a way that meaningfully reflected the patterns observed in the data.

**TABLE 1:** Country categorisation method according to the development of the relative change in the proportion of healthy life years (EU-28, Croatia, Norway, Switzerland, 2005–2019).

Category	Limits of the interval
High expansion	x < ø - (σ * k_1_)
Low expansion	ø - (σ * k_1_) ≤ x ≤ ø - (σ * k_2_)
Dynamic balance	ø - (σ * k_2_) < x < ø + (σ * k_2_)
Low compression	ø + (σ * k_2_) ≤ x ≤ ø + (σ * k_1_)
High compression	ø + (σ * k_1_) < x

Note: x = relative change value, ø = the mean and σ = the standard deviation of the difference in HLYs, for men and women between two years; k_1_ = 1.0; k_2_ = 0.5.

This study focused primarily on the period 2005–2019, i.e., the period immediately prior to the outbreak of the COVID-19 pandemic. Moreover, due to the significant shifts observed during the considered timeframe, we also explored shorter intervals, i.e. 2005–2009, 2010–2014, and 2015–2019. This comprehensive approach allowed for the identification of the distinctive trends in the health of the populations from 2005 to 2019.

## Results

**FIGURE 1:**
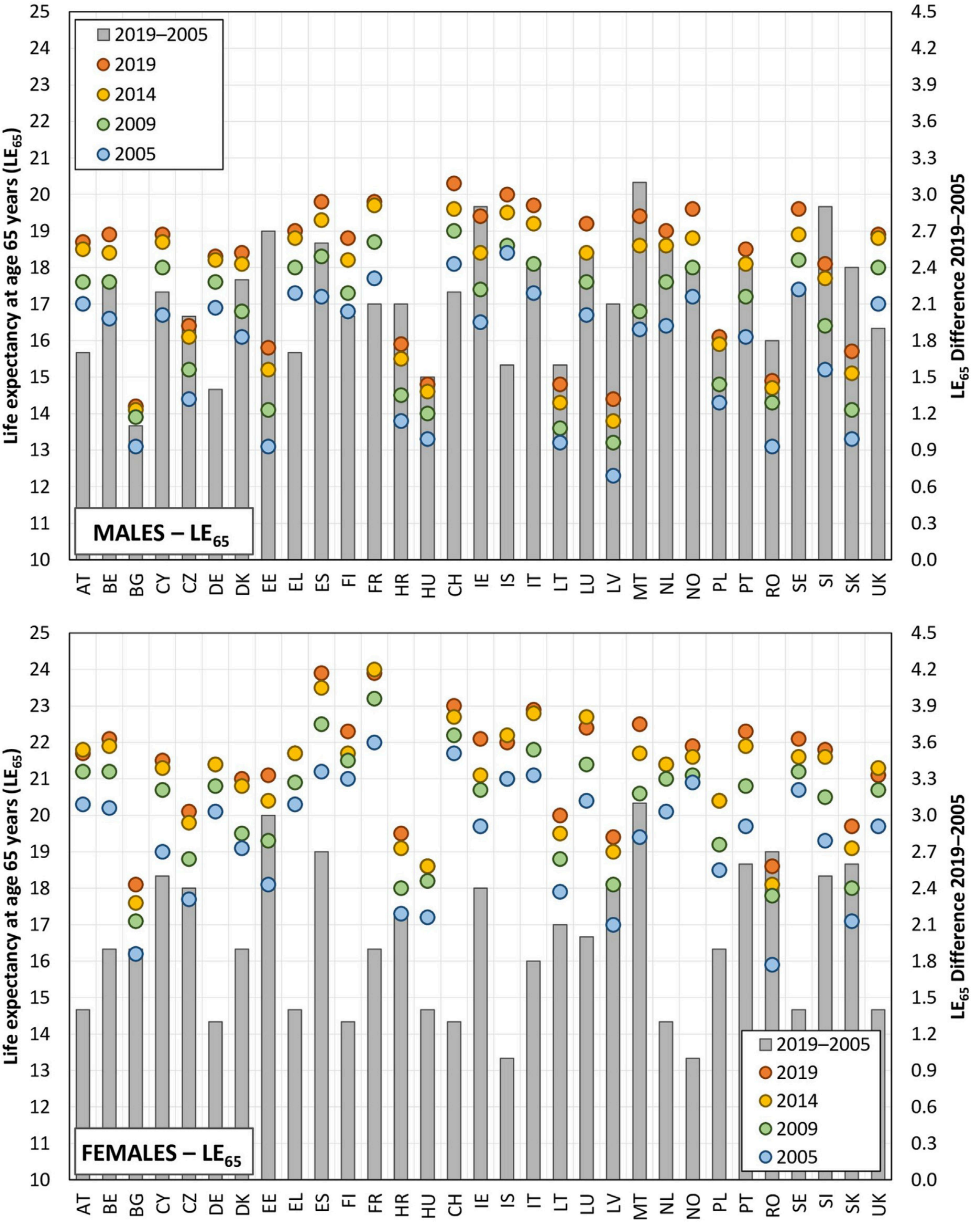
Life expectancy at age 65 years and the difference between the initial and final years of observation (EU-28, Croatia, Norway, Switzerland, 2005, 2009, 2014, and 2019).

The regional picture of differences in life expectancy at age 65 years remained relatively stable throughout the observed period (2005–2019). Certain countries evinced values that exceeded the average value of 19 years for men (Switzerland, Iceland, France) and 22 years for women (France, Spain, Switzerland, Italy), see Figure 1 and others evinced significantly lower average life expectancy at age 65 years, e.g., Latvia, Bulgaria and Lithuania for men and Bulgaria and Romania for women. Figure 1 also illustrates gender-related differences. The average difference in life expectancy between men and women was around 3.5 years across the observed countries in favour of women. Most of the Baltic states were observed to have differences of more than 5.0 years. Conversely, the lowest differences were observed for Iceland and the United Kingdom, i.e., approximately 2.5 years. Although life expectancy at age 65 years increased for both sexes throughout the observed period, the increase was most pronounced in the first two-thirds of the period, i.e. 2005 to 2009 and 2009–2014. Moreover, the life expectancy of women even decreased slightly in the period 2014 to 2019 in a number of countries (most notably Lithuania −0.3 years, followed by Iceland and the United Kingdom −0.2 years and France and Austria −0.1 years).

**FIGURE 2:**
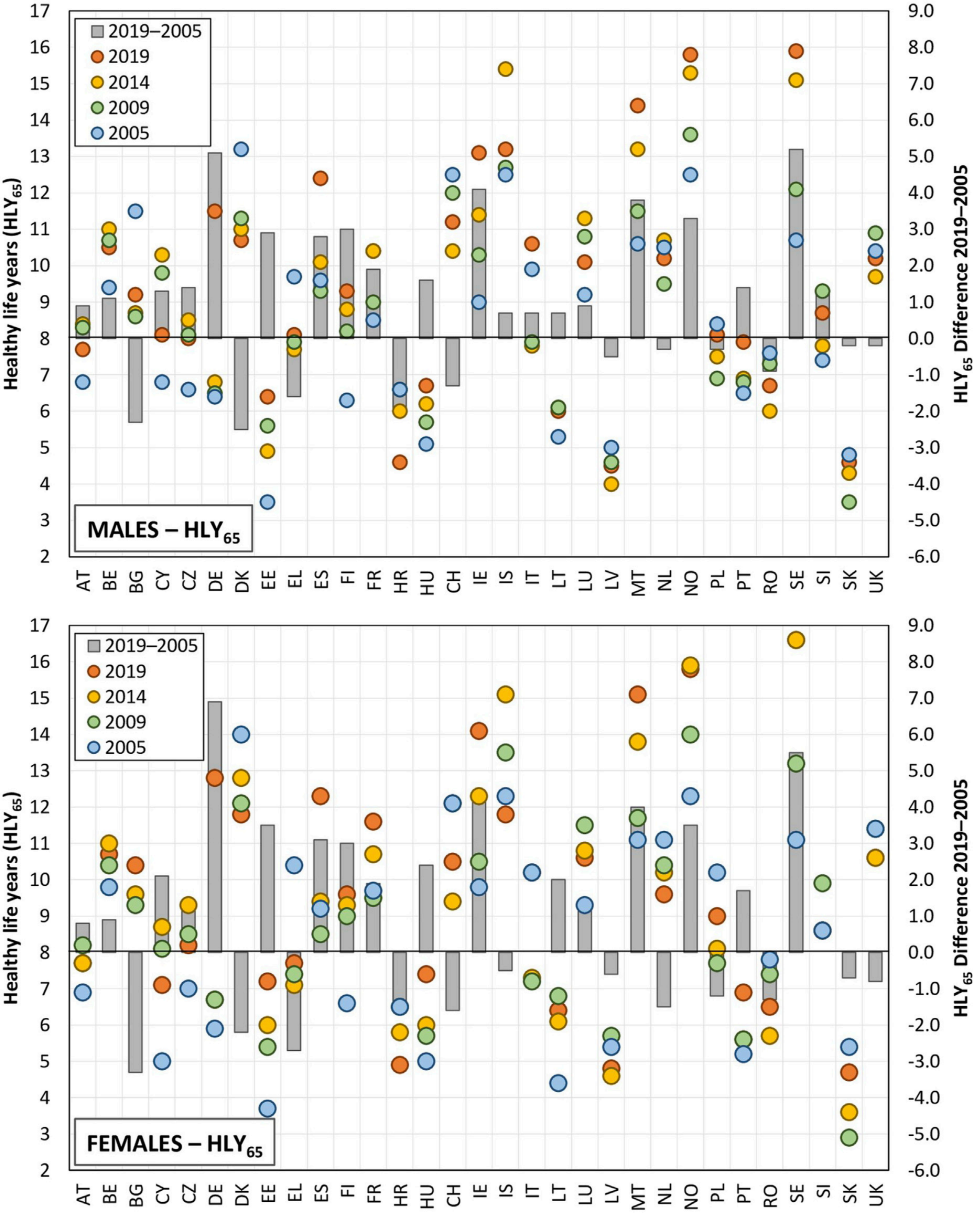
Healthy life years at age 65 years and the difference between the initial and final years of observation (EU-28, Croatia, Norway, Switzerland, 2005, 2009, 2014, 2019).

The evaluation of the average number of HLYs at age 65 years revealed significant differences between the countries in the period 2005–2019. Concerning men, the value of this indicator exceeded 13.5 years in Norway, Sweden and Iceland. In contrast, the average number of HLYs at age 65 years in Slovakia and Lithuania did not even reach 5 years (3.9 and 4.5 years, respectively). Similar regional differences were also identified for women, the average values exceeded 14.3 years in Norway and Sweden, while in contrast, the average value in Slovakia was just 3.8 years (see Figure 2). However, whereas a significant difference was observed in the life expectancy at age 65 years values between women and men (significantly in favour of women), this was not the case of the HLY indicator at age 65 years; the average difference across the 31 countries investigated stood at just 0.2 years. This value was slightly higher for women in 19 of the countries studied (the highest values related to Bulgaria at 1.2 years and Denmark at 1.0 years) and slightly higher for men in 12 of the countries, i.e., the difference between women and men attained negative values, the highest values were noted for Cyprus at −1.4 years and Portugal at −1.2 years).

The simple visual comparison of the data in Figure 2 reveals significant variations in both the HLY and life expectancy trends over the considered cross-sectional years, thus indicating that changes in health indicators are not consistent over time and may vary significantly from one period to another. In addition, different trajectories were observed across countries. For instance, we observed a continuous decrease in the values across all the cross-sectional years for men in Denmark, i.e., from 13.2 years in 2005 to 10.7 years in 2019, whereas we observed firstly a gradual and then a significant increase in the values for men in Germany from the original 6.4 years in 2005 to 11.5 years in 2019. Concerning women, the Netherlands evinced a continuous decrease in the observed values (from 11.1 years to 9.6 years), whereas a continuous increase in values from 11.1 to 15.1 years in 2019 was observed for Malta. The degree of variability for both the trends and the regional differences was found to be significantly higher for HLYs at age 65 years than for life expectancy, which was due mainly to the former reflecting the results of the subjective assessment of health as reported in the sample survey. Such assessments are significantly influenced by current external influences that are not reflected in predominantly stable death rate patterns.

**FIGURE 3:**
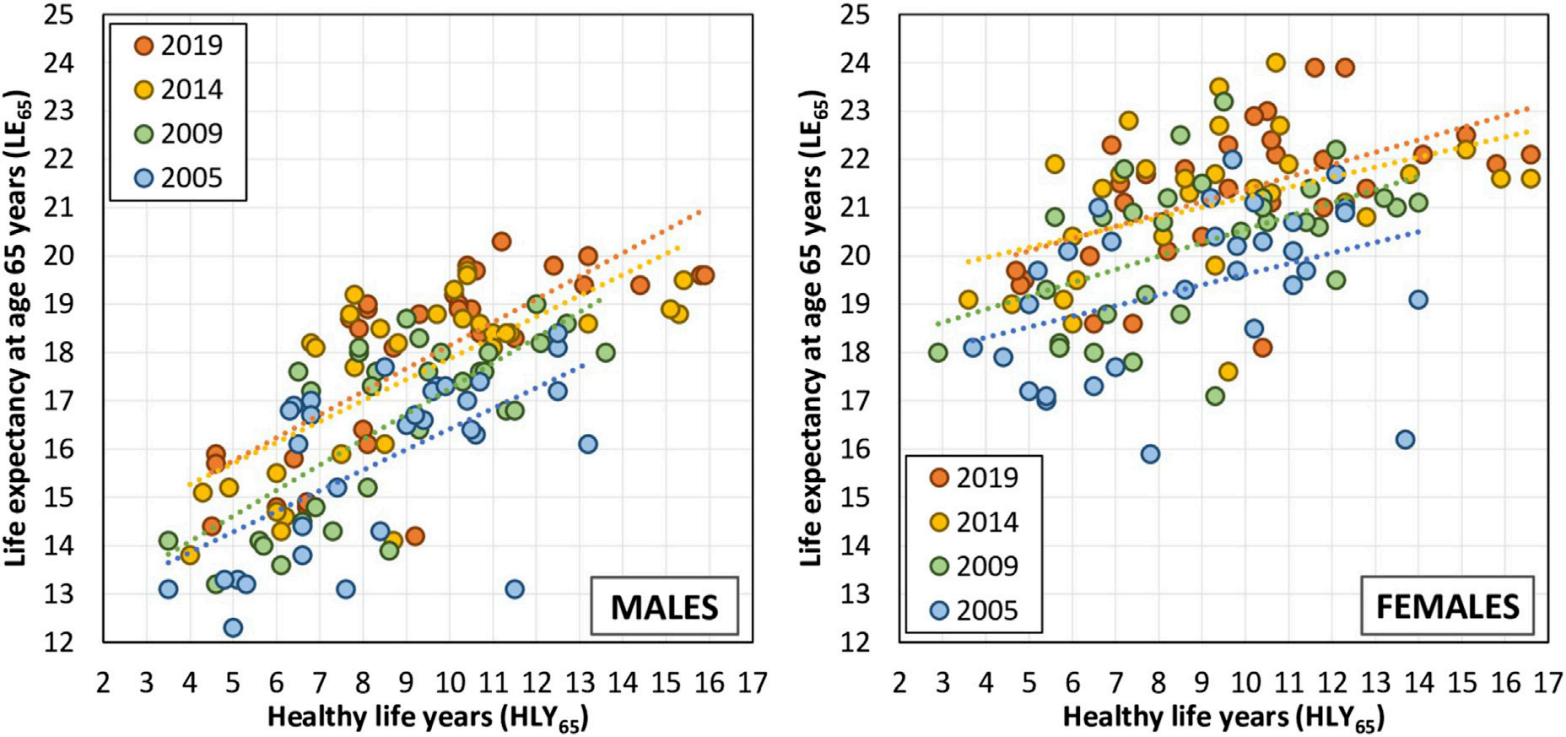
Relationship between the life expectancy indicators at age 65 years and healthy life years at age 65 years (EU-28, Croatia, Norway, Switzerland, 2005, 2009, 2014, and 2019).

Differences were observed in terms of the pace of development between both countries and men and women. The gender comparison (Figure 3) revealed a closer relationship between the increase in life expectancy and the increase in HLY for men than for women, i.e., the difference in the rate of change of the two indicators over time was narrower for men (the dashed trend lines are closer to an angle of 45°, which, in the case of a square geometric network, implies a uniform development trend). A slightly higher degree of variability in terms of the distribution of points across the years can be observed for women.

These findings demonstrate the rather complex nature of the mutual evaluation of life expectancy and HLYs in both time and space. Based on these findings, we subsequently proposed a typology of countries according to the polarity of morbidity expansion versus compression with dynamic balance in the centre of the range. Table 2 describes the basic characteristics (mean, standard deviation) of the share of life spent in good health at age 65 years that determined the type of morbidity considered in the given period and the intervals for the categorisation of countries according to the typology.

**TABLE 2:** Mean, standard deviation and intervals for the categorisation of countries into the country typology according to population ageing theories (EU-28, Croatia, Norway, Switzerland, 2005–2019).

	2005–2009	2010–2014	2015–2019	2005–2019
Males				
Mean	−1.13	−0.43	0.87	−0.97
Standard deviation	8.95	5.02	4.91	11.57
High expansion	<−25.92; −10.08)	<−8.64; −5.45)	<−13.49; −4.04)	<23.84; −12.54)
Low expansion	<−10.08; −5.61)	<−5.45; −2.94)	<-4.04; −1.59)	<−12.54; −6.76)
Dynamic balance	<−5.61; 3.34)	<−2.94; 2.08)	<−1.59; 3.33)	<−6.76; 4.81)
Low compression	<3.34; 7.82)	<2.08; 4.59)	<3.33; 5.79)	<4.81; 10.60)
High compression	<7.82; 13.73>	<4.59; 13.23>	<5.79; 12.63>	<10.60; 24.97>
Females				
Mean	−1.21	−0.31	2.19	0.21
Standard deviation	9.61	5.23	5.16	12.32
High expansion	<−30.18; −10.82)	<−12.81; −5.54)	<17.26; −2.97)	<27.11; −12.11)
Low expansion	<−10.82; −6.02)	<−5.54; −2.93)	<−2.97; −0.39)	<−12.11; −5.95)
Dynamic balance	<−6.02; 3.59)	<−2.93; 2.30)	<−0.39; 4.77)	<−5.95; 6.37)
Low compression	<3.59; 8.39)	<2.30; 4.91)	<4.77; 7.35)	<6.37; 12.53)
High compression	<8.39; 12.81>	<4.91; 13.64>	<7.35; 12.77>	<12.53; 30.46>

**FIGURE 4:**
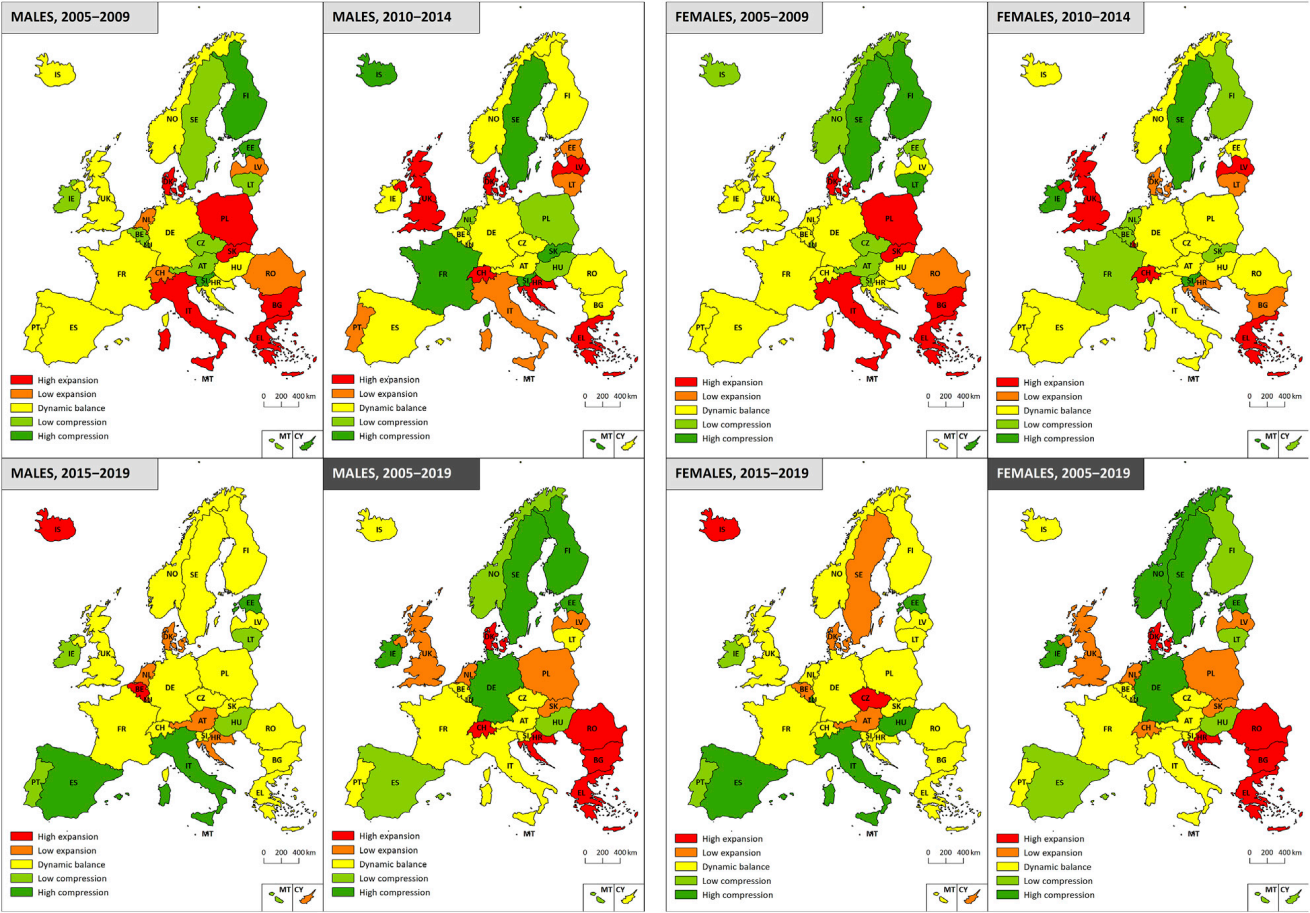
Typology of countries according to population ageing theories (EU-28, Croatia, Norway, Switzerland, 2005–2019).

Figure 4 illustrates the typology of the countries according to the relationship between morbidity and mortality for men and women. A significant change in the types was observed for men over the studied time period. The first sub-period (2005–2009) was characterised by a high degree of heterogeneity on both sides of the imaginary spectrum (10 countries in the morbidity expansion category, 9 in the dynamic equilibrium category and 12 in the morbidity compression category). This pattern partially continued in to the second sub-period (2010–2014) (a ratio of 10-12-9, respectively), although with differing regional trend. For example, the United Kingdom moved from the dynamic equilibrium to a high degree of expansion category, while Slovakia moved from the high degree of morbidity expansion to the high degree of morbidity compression category. The third period (2015–2019) was dominated by the dynamic equilibrium category (almost half of all the countries investigated). A reduction in the representation of countries was observed for both the morbidity expansion and compression categories, especially with concern to the highest morbidity values. Thus, the sub-period data indicated a relatively unstable regional pattern in terms of both time and space. However, the consideration of the whole of the period highlighted certain areas that exhibited common characteristics. The comparison of the beginning and the end of the period 2005–2019 revealed the emergence of the south-eastern margins of the European Union (Romania, Bulgaria, Greece), as well as Switzerland and Denmark, as high morbidity expansion areas, whereas the Nordic countries, together with Germany, Ireland and Spain emerged as high morbidity compression countries.

Similar trends and regional patterns were determined for women. Concerning the initial two relatively heterogeneous sub-periods (the ratios of the countries in the morbidity expansion-dynamic balance-compression categories were 7-13-11 and 9-12-10, respectively) a significant shift of certain countries across the categories were observed, e.g., the United Kingdom and Slovakia, as was the case for men, Italy (high expansion-dynamic balance) and Lithuania (high compression-low expansion). The third sub-period (2015–2019) evinced a relatively homogeneous pattern: the southern part of Europe experienced primarily morbidity compression and the rest of Europe mainly dynamic balance; only Iceland and Czechia differed significantly, i.e., they moved from the low compression category through dynamic balance and into the high morbidity expansion category during the final monitored sub-period. The resulting regional typology for women over the whole of the 2005–2019 period was very similar to that observed for men. Romania, Bulgaria, Greece and Croatia exhibited high morbidity expansion, whereas the Nordic countries, together with Germany, Spain and Ireland fell into the morbidity compression category.

## Discussion

### Summary of Main Findings

The tracking of life expectancy and HLY trends at age 65 years between 2005 and 2019 revealed significant variations in both indicators between the studied countries. Although the differences between men and women were consistent in terms of life expectancy at age 65 years across all the European countries, the gender gap in terms of HLYs at age 65 years was more diverse. Concerning life expectancy, which reflects the mortality patterns in populations, women evince higher values than men, an average difference of around 3.5 years. In general, the lowest values of life expectancy at age 65 years for women match the highest level of this indicator for men, which can be explained by the higher rate of mortality for men over the entire life-span. Although HLYs at age 65 years were found to vary across European countries in a similarly way to life expectancy, the gender gap manifested itself differently. The analysis of the HLYs values revealed that women have higher values in 19 of the countries surveyed and men have higher values than women in 12 of the countries considered.

The 31 European countries were categorized based on the type of morbidity, i.e., compression, expansion or dynamic equilibrium according to which we formulated a typology. We estimated that with respect to men, 11 countries evinced morbidity expansion, 9 countries morbidity compression and the remaining 11 countries dynamic equilibrium over the period 2005–2019 (see Figure 4). The situation was similar for women, i.e. 11, 11 and 9 countries, respectively (see Figure 4). In general, northern European countries tend more towards the compression and eastern European countries towards the expansion of morbidity. When divided into shorter sub-periods, changes were observed in the type of morbidity patterns for several countries in the period 2005–2019, which reflected the extent to which economic and social changes exerted impacts on the health of the population in several countries.

### Limitations

This study should be seen in the light of several limitations. The primary concern is that HLY is a subjective indicator, the assessment of which depends on the self-reporting of the state of health, including activity limitation considerations. This means that perceptions of health may be shaped significantly by various factors, including gender, cultural norms, access to healthcare and socioeconomic status, which act to potentially skew the true representation of the morbidity burden in the population [28]. It is important to note that the definition of HLY used in this research remained consistent throughout the study. However, variations in the perception of health status across different populations has the potential to influence the outcomes. Despite these challenges, the HLY indicator is calculated using data collected annually applying a consistent methodology across most of the European countries. This uniform approach served to reinforce our confidence in HLY as a valuable indicator for comparing health status, both over time and across different European nations.

In addition, the observed heterogeneity in terms of HLYs between different populations may, to a certain extent, depend on differences in the perception of health across European countries. The fact that HLY is a subjective measure can also be seen as a strength rather than a limitation. The self-perception of the state of health provides valuable insight into how individuals evaluate the quality of their lives, which allows for a more nuanced understanding of wellbeing since it captures personal experiences that may not be reflected in objective health statistics.

Aimed at addressing potential limitations associated with the method applied to create the country typology, we employed a standardised approach based on the distance from the mean using the standard deviation. Although other methods were considered, this approach was chosen due to its ability to provide clear distinctions between the considered morbidity scenarios. We acknowledge that alternative methods may have yielded slightly different classifications; however, this method was selected for its robustness and ease of interpretation. Moreover, we ensured that the data was applied consistently across all the countries and periods so as to minimise potential bias in the classification.

In view of the significant impact of the COVID-19 pandemic on public health and society in general, the conducting of a similar analysis using more recent data to assess how the pandemic influenced morbidity trends would be a very valuable exercise. Such a study would provide insight into the shifts that occurred in population health patterns as reflected in the typology of European countries according to the ageing theories – the compression, expansion or dynamic equilibrium of morbidity. Such an analysis would reveal the long-term effects of the pandemic on health and life expectancy and provide valuable information for the formulation of health policy and healthcare planning. Our study, however, aimed to describe morbidity patterns prior to the outbreak of, and disruption caused by, the pandemic.

### Interpretation

Our findings are in line with previous studies that revealed slowdown in increases in life expectancy in recent years in many European countries [19]. In terms of gender differences, we determined higher life expectancy for females consistently across all the European countries studied, as reported previously [29]. Although women have a higher life expectancy than men, the gender gap narrows over time, which suggests mortality convergence between men and women [30], and which is attributable to changes in lifestyle, particularly with concern to women. As women increasingly adopt lifestyle behaviour traditionally associated with men, such as smoking or heavy drinking, their mortality rates have begun to converge with those of men [31].

By evaluating HLYs, the concept of higher male mortality is not universally applied. Of the 31 European countries investigated, higher HLYs at age 65 years were determined for women in 19 countries (primarily in Bulgaria and followed by Denmark, Ireland, France, Sweden, Poland, United Kingdom, Czechia, Finland, Estonia, Germany, Slovenia, Norway, Latvia, Malta, Belgium, Lithuania, Hungary and Luxembourg) and lower HLYs in 12 countries (Croatia, Austria, Netherlands, Slovakia, Switzerland, Iceland, Romania, Greece, Spain, Italy, Portugal and, primarily, in Cyprus), which suggests significantly different morbidity patterns for men and women across Europe, whereas, intuitively, the HLY expectation at age 65 years should be higher for women than men in line with their higher life expectancy.

This study was based on traditional indicators that measure average longevity and average functional limitation; however, a number of recent studies have gone beyond this simple functional limitation/disability approach and focused on the age-at-morbidity onset distribution via the calculation of healthy lifespan inequality indicators [32], the use of hospital admissions data [33] or the incorporation of a broader view that considers the psychosocial and stress burden [34]. The methodology applied clearly exerts an impact on the selected scenarios. For instance, if morbidity is measured by age at the onset of disease, the morbidity burden in Spain increases, thus indicating morbidity expansion [35], which is in contrast to our results that pointed to morbidity compression when applying traditional indicators that measure average longevity and functional limitations. A study by Chen et al. [36] investigated the influence of income distribution in society on changes in health status. They found that countries with higher income inequality tend to experience morbidity expansion and concluded that measures that reduce income inequality serve to promote morbidity compression.

HLY is an indicator that is based on perceived health status, concerning which women tend to evaluate their health status more pessimistically than men due to their more frequent visits to the general practitioner [37] and consequently better information on their health status [38], despite their higher life expectancy. This contrast between self-reported health status and mortality patterns results in lower female HLY rates in proportion to life expectancy values than men [39–41]. These marked differences in terms of the morbidity patterns of men and women during the period 2005 and 2019 suggests that the overall health of the population may also be driven by access to healthcare services and by gender-specific attitudes to illness prevention [42], disease treatment [43] and overall health perceptions [44, 45]. However, the observed gender differences in terms of health status cannot simply be attributed to differences in perceptions and attitudes with concern to the healthcare system. The economic, political and cultural contexts of the country impact men and women differently. Historically, many societies have assigned different roles to men and women, often positioning men in the public and economic sectors and women in domestic and caregiving roles, which potentially affects access to education, employment and leadership positions [46]. Thus, women often face higher risk of poverty due to lower chances of success in the labour market, which potentially results in a greater health burden at higher ages than men [47]. Further, the distribution of diseases and adverse health conditions varies by gender, i.e., women are at higher risk of non-life-threatening conditions and mental health issues [48].

The financial costs associated with treating chronic diseases are extremely high, and given that life expectancy is increasing, chronic diseases will continue to exert pressure on national budgets [49]. It is, thus, essential that the contribution of lifestyle risk factors to chronic diseases is assessed since HLY are affected by a range of aspects including socioeconomic position, healthcare quality, health behaviour, living environment, etc.

### Conclusion

We identified significant variations in healthy life years trends across the studied European countries. The findings highlight the importance of preventive measures aimed at avoiding the deterioration of the health of the population in terms both of the higher frequency of disease-related complications and excessive healthcare costs. It is crucial that policymakers assess the economic and social conditions that impact the health of the population over short periods since long-term trends may act to hide the health consequences of societal changes.

## References

[1] Walshe K McKee M McCarthy M Groenewegen P Hansen J Figueras J Health Systems and Policy Research in Europe: Horizon 2020. Lancet. (2013) 382(9893):668–9. 10.1016/S0140-6736(12)62195-3 23515143

[2] Budych K Karle CA Helms TM. Perspectives on Europe’s Health Care Systems: Meeting Future Challenges through Innovative Health Care Strategies. EPMA J (2014) 5(S1):A82. 10.1186/1878-5085-5-s1-a82

[3] Bongaarts J. How Long Will We Live? Popul Dev Rev (2006) 32(4):605–28. 10.1111/j.1728-4457.2006.00144.x

[4] Wilmoth JR. Demography of Longevity: Past, Present, and Future Trends. Exp Gerontol (2000) 35(9–10):1111–29. 10.1016/s0531-5565(00)00194-7 11113596

[5] Doblhammer G Kytir J. Compression or Expansion of Morbidity? Trends in Healthy-Life Expectancy in the Elderly Austrian Population Between 1978 and 1998. Soc Sci Med (2001) 52(3):385–91. 10.1016/s0277-9536(00)00141-6 11330773

[6] Fries JF. Aging, Natural Death, and the Compression of Morbidity. N Engl J Med (1980) 303(3):130–5. 10.1056/NEJM198007173030304 7383070

[7] Fries JF. Measuring and Monitoring Success in Compressing Morbidity. Ann Intern Med (2003) 139(5 Pt 2):455–9. 10.7326/0003-4819-139-5_part_2-200309021-00015 12965976

[8] Fries JF. The Compression of Morbidity: Near or Far? Milbank Q (1989) 67(2):208–32. 10.2307/3350138 2698444

[9] Fries JF Bruce B Chakravarty E. Compression of Morbidity 1980–2011: A Focused Review of Paradigms and Progress. J Aging Res (2011) 2011:261702–10. 10.4061/2011/261702 21876805 PMC3163136

[10] Gruenberg EM. The Failures of Success. 1977. Milbank Q (2005) 83(4):779–800. 10.1111/j.1468-0009.2005.00400.x 16279967 PMC2690285

[11] Kramer M. The Rising Pandemic of Mental Disorders and Associated Chronic Diseases and Disabilities. Acta Psychiatr Scand (1980) 62(S285):382–97. 10.1111/j.1600-0447.1980.tb07714.x 7468297

[12] Olshansky SJ Rudberg MA Carnes BA Cassel CK Brody JA. Trading off Longer Life for Worsening Health. J Aging Health (1991) 3(2):194–216. 10.1177/089826439100300205

[13] Manton KG. Changing Concepts of Morbidity and Mortality in the Elderly Population. Milbank Mem Fund Q Health Soc (1982) 60(2):183–244. 10.2307/3349767 6919770

[14] Graham P Blakely T Davis P Sporle A Pearce N. Compression, Expansion, or Dynamic Equilibrium? The Evolution of Health Expectancy in New Zealand. J Epidemiol Community Health (2004) 58(8):659–66. 10.1136/jech.2003.014910 15252068 PMC1732857

[15] Klar MK Geyer S Safieddine B Tetzlaff F Tetzlaff J Sperlich S. Trends in Healthy Life Expectancy Between 2002 and 2018 in Germany - Compression or Expansion of Health-Related Quality of Life (HRQOL)? SSM - Popul Heal (2021) 13:100758. 10.1016/j.ssmph.2021.100758 PMC7937823 33732863

[16] Steensma C Loukine L Choi BC. Evaluating Compression or Expansion of Morbidity in Canada: Trends in Life Expectancy and Health-Adjusted Life Expectancy From 1994 to 2010. Heal Promot Chronic Dis Prev Can Res Pol Pract (2017) 37(3):68–76. 10.24095/hpcdp.37.3.02 PMC5602161 28273034

[17] Kreft D Doblhammer G. Expansion or Compression of Long-Term Care in Germany Between 2001 and 2009? A Small-Area Decomposition Study Based on Administrative Health Data. Popul Health Metr (2016) 14:24. 10.1186/s12963-016-0093-1 27418881 PMC4944474

[18] Majewska J Trzpiot G. The Transition in Health in a Population Aged 65 Years and over in Europe. Commun Stat Case Stud Data Anal Appl (2020) 6(1):3–18. 10.1080/23737484.2019.1613456

[19] Welsh CE Matthews FE Jagger C. Trends in Life Expectancy and Healthy Life Years at Birth and Age 65 in the UK, 2008-2016, and Other Countries of the EU28: An Observational Cross-Sectional Study. Lancet Reg Heal Eur (2021) 2:100023. 10.1016/j.lanepe.2020.100023 PMC8042672 33870247

[20] Newton JN. Trends in Health Expectancies across Europe: Countries That Are Achieving Compression of Morbidity and Those That Are Not. Lancet Reg Heal Eur (2021) 3:100078. 10.1016/j.lanepe.2021.100078 PMC8454758 34557806

[21] Eurostat. Database (2022). Available from: https://ec.europa.eu/eurostat/data/database (Accessed January 21, 2022).

[22] Eurostat. Mortality (National Level) (Demo_mor) (2024). Available from: https://ec.europa.eu/eurostat/cache/metadata/en/demo_mor_esms.htm (Accessed August 18, 2024).

[23] Jagger C Robine JM. Healthy Life Expectancy. In: Rogers RG Crimmins EM, editors. International Handbook of Adult Mortality International Handbooks of Population. Dordrecht: Springer (2011). p. 551–68.

[24] Sullivan DF. A Single Index of Mortality and Morbidity. HSMHA Health Rep (1971) 86(4):347–54. 10.2307/4594169 5554262 PMC1937122

[25] Imai K Soneji S. On the Estimation of Disability-Free Life Expectancy. J Am Stat Assoc (2007) 102(480):1199–211. 10.1198/016214507000000040 26279593 PMC4533834

[26] European Commission. European Health Interview Survey (EHIS Wave 2) – Methodological Manual. Report No.: KS-RA-13-018. Luxembourg (2013). Available from: https://ec.europa.eu/eurostat/web/products-manuals-and-guidelines/-/KS-RA-13-018 (Accessed February 7, 2023).

[27] Eurostat. Healthy Life Years by Sex (From 2004 Onwards) (Hlth_hlye) (2022). Available from: https://ec.europa.eu/eurostat/cache/metadata/en/hlth_hlye_esms.htm (Accessed January 21, 2022).

[28] Kurtinová O. Self-perceived Health in the Czech Population: Recent Evidence. Cent Eur J Public Health (2015) 23(1):45–53. 10.21101/cejph.a3996 26036098

[29] Luy M Gast K. Do Women Live Longer or Do Men Die Earlier? Reflections on the Causes of Sex Differences in Life Expectancy. Gerontology (2014) 60(2):143–53. 10.1159/000355310 24296637

[30] Sundberg L Agahi N Fritzell J Fors S. Why Is the Gender Gap in Life Expectancy Decreasing? The Impact of Age- and Cause-Specific Mortality in Sweden 1997–2014. Int J Public Health (2018) 63(6):673–81. 10.1007/s00038-018-1097-3 29654335 PMC6015620

[31] Wensink M Alvarez J-A Rizzi S Janssen F Lindahl-Jacobsen R. Progression of the Smoking Epidemic in High-Income Regions and its Effects on Male-Female Survival Differences: A Cohort-By-Age Analysis of 17 Countries. BMC Public Health (2020) 20(1):39. 10.1186/s12889-020-8148-4 31924192 PMC6954612

[32] Permanyer I Villavicencio F Trias-Llimós S. Healthy Lifespan Inequality: Morbidity Compression From a Global Perspective. Eur J Epidemiol (2023) 38(5):511–21. 10.1007/s10654-023-00989-3 37027116 PMC10080172

[33] Seaman R Höhn A Lindahl-Jacobsen R Martikainen P van Raalte A Christensen K. Rethinking Morbidity Compression. Eur J Epidemiol (2020) 35(5):381–8. 10.1007/s10654-020-00642-3 32418023 PMC7250949

[34] Beltrán-Sánchez H Razak F Subramanian SV. Going Beyond the Disability-Based Morbidity Definition in the Compression of Morbidity Framework. Glob Health Action (2014) 7(1):24766. 10.3402/gha.v7.24766 25261699 PMC4176669

[35] Walter S Beltrán-Sánchez H Regidor E Gomez-Martin C del-Barrio JL Gil-de-Miguel A No Evidence of Morbidity Compression in Spain: A Time Series Study Based on National Hospitalization Records. Int J Public Health (2016) 61(7):729–38. 10.1007/s00038-016-0829-5 27233641 PMC7446746

[36] Chen H Ning J Hu H He H. Distribution of the Compression and Expansion of Morbidity in 194 Countries and Territories, 1990–2016: The Role of Income Inequality. Sociol Health Illn (2023) 45(7):1523–40. 10.1111/1467-9566.13645 37052335

[37] Hunt K Adamson J Hewitt C Nazareth I. Do Women Consult More Than Men? A Review of Gender and Consultation for Back Pain and Headache. J Health Serv Res Policy (2011) 16(2):108–17. 10.1258/jhsrp.2010.009131 20819913 PMC3104816

[38] Warner D Procaccino JD. Toward Wellness: Women Seeking Health Information. J Am Soc Inf Sci Technol (2004) 55(8):709–30. 10.1002/asi.20016

[39] Case A Paxson C. Sex Differences in Morbidity and Mortality. Demography (2005) 42(2):189–214. 10.1353/dem.2005.0011 15986983

[40] Idler EL. Discussion: Gender Differences in Self-Rated Health, in Mortality, and in the Relationship Between the Two. Gerontologist (2003) 43(3):372–5. 10.1093/geront/43.3.372

[41] Benyamini Y Leventhal EA Leventhal H. Gender Differences in Processing Information for Making Self-Assessments of Health. Psychosom Med (2000) 62(3):354–64. 10.1097/00006842-200005000-00009 10845349

[42] Deeks A Lombard C Michelmore J Teede H. The Effects of Gender and Age on Health Related Behaviors. BMC Public Health (2009) 9:213. 10.1186/1471-2458-9-213 19563685 PMC2713232

[43] Vlassoff C. Gender Differences in Determinants and Consequences of Health and Illness. J Health Popul Nutr (2007) 25(1):47–61. 17615903 PMC3013263

[44] Verbrugge LM. Multiple Roles and Physical Health of Women and Men. J Health Soc Behav (1983) 24(1):16–30. 10.2307/2136300 6853995

[45] Ross CE Bird CE. Sex Stratification and Health Lifestyle: Consequences for Men’s and Women’s Perceived Health. J Health Soc Behav (1994) 35(2):161–78. 10.2307/2137363 8064123

[46] Guberman N Maheu P Maille C. Women as Family Caregivers: Why Do They Care? Gerontologist (1992) 32(5):607–17. 10.1093/geront/32.5.607 1427272

[47] Bastos A Casaca SF Nunes F Pereirinha J. Women and Poverty: A Gender-Sensitive Approach. J Socio Econ (2009) 38(5):764–78. 10.1016/j.socec.2009.03.008

[48] Riecher-Rössler A. Sex and Gender Differences in Mental Disorders. The Lancet Psychiatry (2017) 4(1):8–9. 10.1016/S2215-0366(16)30348-0 27856397

[49] Brennan P Perola M van Ommen G-J Riboli E, European Cohort Consortium. Chronic Disease Research in Europe and the Need for Integrated Population Cohorts. Eur J Epidemiol (2017) 32(9):741–9. 10.1007/s10654-017-0315-2 28986739 PMC5662668

